# Introduction of a qualitative perinatal audit at Muhimbili National Hospital, Dar es Salaam, Tanzania

**DOI:** 10.1186/1471-2393-9-45

**Published:** 2009-09-19

**Authors:** Hussein L Kidanto, Ingrid Mogren, Jos van Roosmalen, Angela N Thomas, Siriel N Massawe, Lennarth Nystrom, Gunilla Lindmark

**Affiliations:** 1 Department of Obstetrics and Gynaecology, Muhimbili University of Health and Allied Sciences, Dar es Salaam, Tanzania; 2 Department of Obstetrics and Gynaecology, Muhimbili National Hospital, Dar es Salaam, Tanzania; 3 Department of Public Health and Clinical Medicine, Epidemiology and Public Health Sciences, Umeå University, Umeå, Sweden; 4 Department of Obstetrics, Leiden University Medical Centre and Section of Health care and Culture, VU University Medical Centre, Amsterdam, The Netherlands; 5 Department of Clinical Science, Obstetrics and Gynaecology, Umeå University, Umeå, Sweden; 6 Department of Women's and Children's Health, Academic Hospital, Uppsala, Sweden

## Abstract

**Background:**

Perinatal death is a devastating experience for the mother and of concern in clinical practice. Regular perinatal audit may identify suboptimal care related to perinatal deaths and thus appropriate measures for its reduction. The aim of this study was to perform a qualitative perinatal audit of intrapartum and early neonatal deaths and propose means of reducing the perinatal mortality rate (PMR).

**Methods:**

From 1^st ^August, 2007 to 31^st ^December, 2007 we conducted an audit of perinatal deaths (n = 133) with birth weight 1500 g or more at Muhimbili National Hospital (MNH). The audit was done by three obstetricians, two external and one internal auditors. Each auditor independently evaluated the cases narratives. Suboptimal factors were identified in the antepartum, intrapartum and early neonatal period and classified into three levels of delay (community, infrastructure and health care). The contribution of each suboptimal factor to adverse perinatal outcome was identified and the case graded according to possible avoidability. Degree of agreement between auditors was assessed by the kappa coefficient.

**Results:**

The PMR was 92 per 1000 total births. Suboptimal factors were identified in 80% of audited cases and half of suboptimal factors were found to be the likely cause of adverse perinatal outcome and were preventable. Poor foetal heart monitoring during labour was indirectly associated with over 40% of perinatal death. There was a poor to fair agreement between external and internal auditors.

**Conclusion:**

There are significant areas of care that need improvement. Poor monitoring during labour was a major cause of avoidable perinatal mortality. This type of audit was a good starting point for quality assurance at MNH. Regular perinatal audits to identify avoidable causes of perinatal deaths with feed back to the staff may be a useful strategy to reduce perinatal mortality.

## Background

The perinatal mortality rate (PMR) in Tanzania is among the highest in the world. Therefore, to reduce perinatal mortality needs a major effort in order to achieve the Millennium Development Goal no. 4 to reduce child mortality by two third (MDG4). Although the Tanzania Demographic and Health Survey [1] showed a decrease in under - 5- mortality from 147 deaths/1000 in 1994 to 1999 to 112 deaths/1000 in 2000 to 2004, the neonatal mortality rate (32/1000 live births) had not declined. The reduction of child deaths can only be achieved if perinatal survival is improved; several studies have indicated up to half of the perinatal deaths globally occur as a direct consequence of poorly managed deliveries [2-4].

Hospital-based studies in low income countries have shown that 3 out of 4 perinatal deaths may be due to suboptimal care [5,6]. Our previous study in Dar es Salaam 1999-2003 estimated the PMR at 123 per 1000 total births and the majority of these deaths were assumed preventable [6]. Thus, reduction of PMR and improvement of maternal and child health requires identification of service-related factors leading to perinatal deaths [7,8]. One approach is to perform clinical audits in obstetric care, i.e. retrospective critical reviews of clinically undesirable pregnancy-related events.

Perinatal mortality audits in obstetrics are intended to determine primary and final causes of death as well as suboptimal factors and missed opportunities to ascertain how to improve future management. Preventable factors could be health professional related, such as a health provider failing to perform recommended procedures, or be administration related, such as unavailability of necessary drugs, other preventable factors could be patient-related, such as delay to seek medical assistance due to various reasons [9]. The fundamental goal of establishing perinatal audits in areas with high PMR is to reduce the number of perinatal deaths through an improvement in the quality of care. Several studies [10,11] have shown a strong association between the establishment of an effective audit process and improvement of the quality of maternal health services and a reduction of maternal and perinatal foetal mortality rates.

The aim of this study was to introduce a qualitative perinatal audit in an urban tertiary centre in Tanzania, the main focus being on obstetric care during labour and delivery.

## Methods

### Setting

The study was carried out in the labour ward at Muhimbili National Hospital (MNH), a teaching hospital for Muhimbili University College of Health Sciences and one of four large consultant hospitals in the United Republic of Tanzania. It is situated in Dar es Salaam, which has a population of about 2.5 million and an annual population growth rate of 4.3% [1]. The hospital serves as a referral centre for the city of Dar es Salaam and the neighbouring coastal region. Annual number of deliveries was about 10,000, corresponding to about 30 deliveries per day, out of which 80% are low-risk deliveries. Every month there is a perinatal mortality meeting involving all members of the department of obstetrics and representatives from the neonatal unit, where the monthly trend as well as a selected perinatal mortality case is discussed, however not in the form of a formalised audit.

There are three shifts for nurses working in the labour ward, each with six midwives. One specialist obstetrician, one consultant obstetrician and one resident (house officer) are on call every day. After a normal uneventful vaginal delivery the mothers and babies are often observed in hospital for 6-10 hours. During this time the babies also get BCG and polio vaccinations before being discharged. Babies delivered by caesarean section (CS) or those with low Apgar score (<7) were admitted to the neonatal ward, which was just one floor up from the labour ward. The unit also admitted sick babies from other hospitals.

### Material

Information on all perinatal deaths occurring from 1^st ^August, 2007 to 30^th ^December 2007 were collected through case notes, antenatal cards, and maternity midwifery registry records and classified according to the modified Nordic Baltic classification [12]. A check list was used to make sure that all the required information was obtained. For each stillbirth and early neonatal death, information was abstracted on the date of birth, residential area of the woman, antenatal care attendance, maternal age, parity, estimated gestational age, birth weight, sex and vital status of the baby at birth (Apgar score at 1 and 5 minutes), multiple births, and mode of delivery. Women with at least one antenatal care (ANC) visit were considered as having received antenatal care.

Perinatal death was defined according to the World Health Organization (WHO) 1997 definition of viability, i.e. a birth weight of ≥500 grams. However, for the purpose of this paper only babies weighing ≥1500 grams were included in the analysis as the neonatal unit of MNH adequately can take care of this group of newborns. The neonates admitted to the neonatal unit soon after the delivery was followed for 7 days in order to record early neonatal deaths.

### Audit procedure

The audit was performed by obstetricians to focus on the care given during labour and delivery from an obstetric perspective. Cases of stillbirth and early neonatal death weighing ≥1500 grams were assessed through narratives from case notes by an expert panel of two external and one internal auditor. Narratives from the case notes and grading forms were prepared in English by the first author and dispatched to the internal auditor and the two external auditors in the Netherlands and Sweden. The auditor from the Netherlands had worked in Tanzania before and has vast experience on African health systems whereas the auditor from Sweden never had worked in Africa.

The audit protocol was prepared and agreed at a perinatal audit workshop convened at the hospital. This workshop was attended by members of the department i.e. nurses, midwives and doctors as well as the auditors. The role of the audit panel members was to identify those situations that were critical and required action. If the required action was not covered by the objective criteria that were formulated beforehand, it was up to the personal judgment of the panel member to assess the adequacy of action taken and to comment on the level of sub-optimality. Suboptimal factors were identified in the antepartum, intrapartum and neonatal periods, and classified in three levels of delay:

1. *Maternal/social factors *(delay related to the patient or relatives).

2. *Communication/infrastructure *(delay due to transport problem).

3. Health *care factors *(Delay of appropriate care after admission to hospital).

The auditors worked independently using a structured assessment protocol and grading form. The contribution of each suboptimal factor to the fatal outcome was assessed and a final grade (Table 1) was given by each auditor. After collating the assessment forms the coordinator (corresponding author) computerized the information.

**Table 1:** Perinatal audit form for identification of suboptimal factors and grading

**Item**	**Choice**	**Selection**
Level of delay	1 = Maternal social (community level)	
	2 = Infrastructure service organization	
	3 = Care	
	4 = Infrastructure and care	
	5 = All of above	
	6 = No delay	
Contribution of the suboptimal care to the foetal death	1 = Unlikely	
	2 = Possibly	
	3 = Likely	
Final grading	**Grade 0 **-No suboptimal care identified	
	**Grade I **-Suboptimal care, identified but unlikely to have contributed to the fatal outcome, different management would have made no difference to the outcome	
	**Grade II **-Suboptimal care identified and might have contributed to the fatal outcome. Different management might have made a difference to the outcome.	
	**Grade III **-Suboptimal care identified and is likely to have contributed to the fatal outcome. Different management would reasonably be expected to have made a difference to the outcome. A clearly avoidable factor implying that the adverse outcome could have been prevented.	
Overall suboptimal graded 0, 1, II or III according to above definition		
Do you think there was sufficient information available to assign a final grade in this case	1 = Yes 2 = No	
Do you consider likely that this death was preventable?	1 = Yes 2 = No	
Number of panel member..................	Date..............................	

### Statistical analysis

Degree of agreement between auditors was assessed by the kappa coefficient using the shareware Win Pepi. We adopted the Landis and Koch scale [13] i.e. a kappa coefficient of 0-0.20 indicates poor agreement, 0.21-0.40 fair agreement, 0.41-0.60 moderate agreement, 0.61-0.80 good agreement, and 0.81-1 very good agreement.

## Results

**Figure 1:**
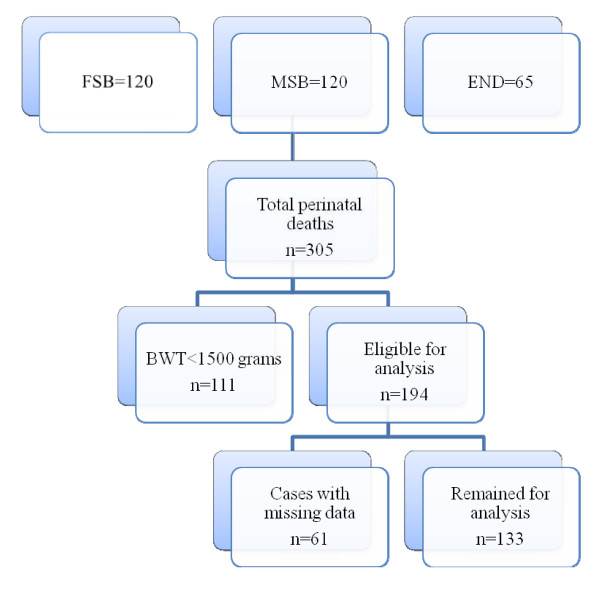
**Cases sorting flow chart**.

During the study period of five months 3767 births were recorded out of which 3449 were live births. There was a total of 240 stillbirth of which 120 cases were macerated pre-labour stillbirth, 120 cases of fresh intrapartum stillbirth and 65 cases of early neonatal death, thirteen cases were unclassified, therefore there were 305 perinatal deaths (Figure 1).

The stillbirth, early neonatal mortality and overall perinatal mortality rates in the material were 64/1000 births, 19/1000 live births and 81/1000 births, respectively. Perinatal deaths were significantly more common among teenage mothers (33% vs. 13%; p < 0.001), and in preterm babies (54% vs. 18%; p < 0.0001; Table 2). However, almost half of the deaths were babies born at term.

**Table 2:** Maternal characteristics of women who experienced perinatal death at Muhimbili National Hospital, August-December 2007

**Maternal characteristic**	**Perinatal deaths**		**All births**	
		
	**n**	**%**	**n**	**%**
**Age (years)**				
13-19	100	32.8	487	13.0
20-29	61	20.0	1086	28.9
30-39	132	43.3	2055	54.7
≥40	12	3.9	126	3.4
**Parity**				
0	121	39.7	1507	40.2
1-2	123	40.3	1689	45.0
≥3	61	20.0	558	14.8
**Gestational age (weeks)**				
28-33	93	30.5	168	4.5
34-36	70	23.0	518	13.8
≥37	142	46.6	3068	81.7
**No of ANC visits**				
0	0	0.0	1	0.0
1-3	155	50.8	1333	35.5
≥4	150	49.2	2420	64.5
**Source of admission**				
Home	153	50.2	762	20.3
Hospital transfer	152	49.8	2992	79.7

Total	305	100	3754	100

For the audit purpose out of 305 perinatal deaths we excluded 111 babies weighing <1500 grams (73 macerated stillbirths, 20 fresh stillbirths, 18 neonatal deaths) as well as all unclassified cases. Finally, we excluded an additional 61 perinatal deaths with missing values or case note (29 macerated stillbirths, 22 fresh stillbirths, 10 neonatal deaths) hence leaving 133 cases for the audit (corresponding to 68.5% of perinatal deaths weighing 1.5 kg or 43.6% of total perinatal deaths during the study period); Figure 1).

Table 3 summarizes the causes of perinatal deaths and maternal medical conditions. One third of the patients were admitted without audible foetal heart beats. Birth asphyxia was the main cause of intrapartum fresh stillbirth (47%) and early neonatal death (51%), whereas eclampsia (25%) anaemia (14%) and preeclampsia (8.3%) were the main maternal medical conditions. The majority of stillbirths were fresh, indicating foetal demise during labour or less than 24 hours before delivery.

**Table 3:** Causes of stillbirths and neonatal deaths weighing ≥1500 grams and maternal medical conditions

**Perinatal deaths**	**Number**	**%**
**Still birth**		
Macerated	18	13.5
Fresh	78	58.7
Admitted with foetal heart beat	32	24.1
Admitted without heart beat	46	34.6
Early neonatal death	37	27.8

Total	133	100

**Intrapartum causes of fresh stillbirth**		
Birth asphyxia	37	47.4
Abruption Placenta	18	23.2
Ruptured uterus	5	6.4
Haemorrhage other than abruption	3	3.8
Congenital anomaly	3	3.8
Cord prolapsed	2	2.6
Unknown	10	12.8

Total	78	100

**Causes of early neonatal death**		
Birth asphyxia	19	51.3
RDS	8	21.6
Meconium aspiration	3	8.1
Neonatal jaundice	3	8.1
Head injury during delivery	3	8.1
Aspiration pneumonitis	1	2.7

Total	37	100

**Maternal medical condition**		
Eclampsia	33	24.8
Anaemia	19	14.3
Preeclampsia	11	8.3
Severe anaemia	6	4.5
HIV/AIDS	5	3.8
Severe malaria	2	1.5
Diabetes mellitus	1	0.8
Others	2	1.8
None	54	41.3

Total	133	100

All three auditors identified suboptimal factors in about 80% of audited cases out of which about 50% were found to be the likely cause of the adverse perinatal outcome (Table 4). The external auditors I and II reported 53% and 44% of the deaths as associated to suboptimal care as compared to 44% for the internal auditor (Table 4). Most cases (37% and 43%) were given a final grade III by external auditors I and II as compared to 26% by the internal auditor. In 22 cases auditors did not find evidence of suboptimal care (15, 19, and 22 cases for external auditors 2, 1 and internal auditor respectively.

**Table 4:** Suboptimal care and its contribution to adverse perinatal outcome, level of delay and final grading by auditors

	**External 1**		**External 2**		**Internal**	
	
	**No**	**%**	**No**	**%**	**No**	**%**
**Suboptimal factor:**						
Yes	105	78.9	115	86.4	107	80.5
No	19	14.3	15	11.3	16	12.0
No opinion	9	6.8	3	2.3	10	7.5
**Contribution of suboptimal factor to adverse outcome:**						
Unlikely	9	6.8	25	18.8	23	17.3
Possibly	30	22.5	35	26.3	49	36.8
Likely	71	53.4	58	43.6	58	43.6
No opinion	23	17.3	15	11.3	3	2.3
**Level of delay:**						
Community/family level	1	0.8	10	7.5	9	6.8
Infrastructure	4	3.0	2	1.5	8	6.0
Care	86	64.6	80	60.1	62	46.6
Infrastructure and care	21	15.8	22	16.6	35	26.3
No comments	21	15.8	19	14.3	19	14.3
**Final grade:**						
0	19	14.3	15	11.3	22	16.5
1	11	8.3	19	14.3	11	8.3
II	54	40.6	42	31.6	65	48.9
III	49	36.8	57	42.9	35	26.3
**Was the information available sufficient to assign a final grade?**						
Yes	116	87.2	120	90.2	114	85.7
No	15	11.3	12	9.0	18	13.5
No opinion	2	1.5	1	0.8	1	0.8
**Do you consider likely that this death was preventable?**						
Yes	101	75.9	69	51.6	105	78.9
No	24	18.0	58	43.6	24	18.0
No opinion	8	6.0	6	4.5	4	3.0
**Avoidable factors detected by auditors:**						
Inadequate foetal heart monitoring during labour	57	42.9	55	41.4	62	46.6
Delayed operation	18	13.5	22	16.5	15	11.3
Delayed referral	20	15.0	18	13.3	31	23.3
Failure to diagnose	4	3.0	10	7.5	7	5.3
Traumatic assisted delivery	2	1.5	1	0.8	1	0.8
Failure to resuscitate a newborn	0	0	0	0	3	2.3
Inadequate antenatal care	3	2.3	2	1.5	3	2.3
Others	10	7.5	17	12.8	4	2.8
None	19	14.3	8	6.0	7	5.3

Total	133	100	133	100	133	100

The external auditors agree that over 75% of audited cases the death was preventable as compared to 52% by the internal auditor. Furthermore, all auditors considered the information provided as sufficient to assign the final grade in over 85% of the cases.

Given that 51.9% of the dead babies had heart beats on arrival and that suboptimal care was assessed as the likely reason for deaths in 43-53% by the auditors, it can be estimated that one out of four deaths could be prevented by improved standard of obstetric care.

The avoidable factors identified by the auditors are presented in Table 4. The deficiencies in clinical care were dominating, mainly the professional performance but also the organisation. Rather few cases of patient related (community) delay were identified.

**Table 5:** Level of agreement among auditors

**Item**	**External 1 vs. External 2**	**External 1 vs. Internal**	**External 2 vs. Internal**
Suboptimal factor (Yes, No)	0.25	0.078	0.19
Contribution of suboptimal factor to adverse outcome	0.30	0.29	0.35
Final grading (0, I, II, III)	0.31	0.31	0.37
Was the information available sufficient to assign a final grade	0.30	0.05	0.05
Do you consider likely that this death was preventable	0.27	0.26	0.34

The two most important areas for improvement of obstetric care were monitoring during labour of the foetal condition and delay of clinical decisions as well as delay of implementation of decided actions. Lack of 24 hour comprehensive emergency obstetric care, and problems with transport from the municipal hospitals contributed to delays in referrals. Furthermore, ongoing hospital renovation at MNH caused severe shortage of theatre space; only one theatre for surgery in obstetrics and gynaecology was available during the study period.

Table 5 summarizes the level of agreement among auditors. On evaluation of the quality of care, there was a better agreement between the external auditors (κ = 0.25) as compared to the external auditors versus the internal auditor (κ = 0.08-0.19). However, there was a better agreement between external auditor no 2 and the internal auditor with respect to final grading and contribution of suboptimal factors to adverse outcome κ = 0.31-0.37 and 0.30-0.35). There was a better agreement between the two external auditors in the questions about the adequacy of the information to assign the final grade and case preventability (κ = 0.30 and κ = 0.25 respectively) as compared to both external and internal auditor.

### Monitoring during labour

Poor foetal heart rate monitoring during labour was indirectly associated with over 40% of perinatal deaths. Among auditors, poor foetal monitoring had 57 (43%), 55 (41%) and 62 (47%) agreement. Poor documentation also was a major problem as has been shown by the exclusion of many cases lacking information. The majority of patients with induction of labour were induced with vaginal misoprostol. The auditors found that the dose used was too high (50 microgram) and there was no clinical assessment of the foetus before induction to determine if the foetus would not be able to endure the induced labour due to severe dysfunction of the placental-foetal unit. This would have increased the risk of asphyxia caused by hyper-stimulation. It was also noted that patients with induced labour were poorly monitored. Partogram were not properly filled and foetal heart rate monitoring was inadequate.

### Delayed management

A proportion of three quarters (n = 23) of patients referred to MNH took a very long time to reach MNH due to transport problems. In order to save fuel ambulances wait until there are more than two patients, furthermore, in municipal hospitals the same ambulance ferries patients from other wards as well, so there was a tendency of waiting irrespective of the degree of emergency. Some of the cases referred for CS could have been delivered at the referring hospital, which might have prevented foetal death.

At MNH, after making a decision to deliver by CS, there were very long intervals between decision and actual procedure. Some patients were delivered vaginally while waiting for CS, and instrumental vaginal delivery could have improved the outcome of these babies. The auditors suggested more frequent use of vacuum extraction (Ventouse) delivery. This procedure might have had decreased the adverse perinatal outcome and the events of vaginal delivery in the theatre for patients scheduled for emergency caesarean section (CS). In case of foetal distress, prompt abdominal delivery and transfer of the baby to the neonatal unit could have improved the outcome for many babies. Prolonged labour diagnosed at MNH as well as at the referring hospitals contributed probably substantially to intrapartum and early neonatal deaths.

Initiation of care at MNH was not immediate after the patient has been admitted. Even patients with seizures were not assessed by doctors within two hours after admission. All auditors concurred that delay in care was a major adverse factor for perinatal outcome.

Other comments by the auditors were related to failure to diagnose as well as to consult with more experienced doctors and this resulted in wrong management. There was often lack of communication between senior and junior doctors.

## Discussion

Previous studies conducted in Tanzania and elsewhere put more emphasis on risk factors and adverse perinatal outcome instead of evaluating what is really happening during labour [14]. The current study is the first attempt at MNH to qualitatively identify potentially avoidable causes of perinatal deaths and suggest course of action to reduce perinatal mortality.

Previous studies have indicated that the majority of perinatal mortality is actually preventable without extra resource inputs. Perinatal audit in the South Western highlands of Tanzania by van Roosmalen [15] resulted in a reduction of perinatal mortality rate from 71 to 39/1000 following the introduction of a new obstetric policy that emphasized the prevention of prolonged labour, the early detection of foetal distress, and better recognition of women with high risk pregnancies.

According to this audit intrapartum deaths and neonatal deaths could be prevented by up to 64% according to external auditors and by 79% by internal auditor. These figures are much higher as compared to a similar study in Sudan [16]. It may be questioned at this point whether this type of qualitative audit improve care on itself. To achieve this prolonged follow-up studies are necessary to measure the impact like what has been done for ten years in Mozambique [17]. This needs resources and may necessitate relinquishing from other quality improvement activities and concentrate on measuring the effect of audit. However, it is ethical and very important to have continuous quality improvements. This study is the beginning of a continuous process to improve care in a hospital where audit was non-existent. This audit focused on adverse perinatal outcome and carries a risk encouraging interventions such as operative deliveries. Therefore, it is imperative that it should be complemented by other quality improvement activities rather than standing alone. However, this kind of audit process provides a good start for quality improvement in developing countries like Tanzania. It is obvious that it is not possible in a busy labour ward like MNH to prepare case notes of all cases for audit, but a good way is to give the task to one of the junior doctors to prepare case stories for assessment e.g. 2-3 cases a week that can be presented and assessed at the weekly meeting. One can alternate between types of cases as the senior obstetrician decides. In general practice it is not the main aim to get statistics of the sort we have in a paper, so there is no need to assess all cases as long as all types of complications are represented.

An audit has a great strength in quality assessment because of the continuous and systematic assessment by peer groups. It enables identification of unique aspects by comparison with established guidelines. Audit can bring change by continuous feedback to stake holders for the purpose of intervention. There are however, diverging opinions about the utility of perinatal audit, some report an effect in decreasing risk of perinatal mortality by improving the practice of health care providers while others claim that benefits have never been adequately evaluated [18]. Audit in poor resource countries like Tanzania is always compromised by the poor quality of information recorded in women's case notes, and by conflict between ideal care and available resources. In this study the poor quality of the patient records including lack of partogram has been identified as a problem. The improvement of recording practices by doctors and nurses is therefore recommended.

The high level of suboptimal care and concurrent avoidable perinatal death noted here is a finding similar to previous studies in Africa [19] but much higher than that found in European studies [20,21]. The current study used the term suboptimal care when there was a departure from acceptable evidence based standards. However, suboptimal care does not necessarily lead to a perinatal death, because it can be identified both in cases with good and adverse outcome. In contrast, avoidable factors always refer to their relationship with adverse outcome. This study graded levels of suboptimal care as an unlikely, possibly or a likely cause of the adverse perinatal outcome. About half of the audited deaths were likely to have been due to suboptimal care, and this indicates that changes in the daily routines can bring significant reduction in perinatal mortality in this urban tertiary centre.

The methodology used had the advantage that the auditors included both a local obstetrician and two external auditors. One of the external auditors had vast experience of obstetric practice in Africa and the other obstetrician had not practiced in Africa. We consider that this combination of auditors added strength to our study and might have minimized bias in the total assessment. The internal auditor was an obstetrician working in the same hospital which might have made her less critical, but she also had the advantage of knowledge of the working environment. It was more common that the external auditors considered that clinical action was delayed than the internal auditor; still, there was overwhelming agreement among auditors that about 80% of the perinatal deaths assessed were characterized by suboptimal care. The evaluation of the cases and grading depended greatly on the individual auditors experience as reflected by fair to poor agreement among auditors. The external auditors were more critical than the internal auditor. It would be questioned as to whether external auditors should always be invited in this kind of audit, no, however, for this particular study it was necessary because it was the introduction of an audit in this hospital with very high perinatal mortality, therefore, it was as well a capacity building process.

Although the level of delay ranged from the patient herself to the health care in the institution, this audit was primarily concerned with the type of service the patient received after arrival in the health institution. This is also the most useful part of an audit, because it is within the institution that change can be instituted immediately. Lack of proper foetal monitoring during labour at MNH has nothing to do with problems in the community or in the referring hospitals.

The main limitations of this study are the inability to include events during the antenatal period and many of the perinatal death due to inadequate documentation. The finding of almost equal numbers of fresh and macerated stillbirths suggests that many problems were linked to insufficient care during the antenatal period. Previous studies done in Tanzania [22,23] and elsewhere [24] revealed that poor antenatal care and infections are associated with antepartum stillbirths. In our material one third of the perinatal deaths were related to eclampsia and preeclampsia that were first diagnosed at admission, a clear indication of inadequate antenatal care. A number of recent studies have questioned the effectiveness of some components of antenatal care in reducing the risk of poor pregnancy outcomes. In particular, questions have been raised on the effectiveness of maternal weight and blood pressure measurements, and a too high frequency of visits [25]. It is, therefore, necessary that the limited resources available target those components of antenatal care proven to be the most cost-effective. When hypertension is diagnosed during ANC, it should be evaluated and followed up; otherwise complications like eclampsia will not be prevented.

Poor documentation is a major bottleneck in most developing countries; in this study many case notes lacked important information like Apgar score, type of stillbirth (Macerated still birth (MSB) or Fresh still birth (FSB)), sex of the baby etc. This necessitated exclusion of these cases from the study; however, since there were a big number of cases for analysis we consider that excluded cases are not a major source of bias.

MNH is the only public referral tertiary hospital in Dar es Salaam, the three municipal hospitals do not have an effective round the clock comprehensive emergency obstetric care, and therefore, most cases which needed emergency operative delivery and blood transfusion were referred to MNH where there was a long queue for operation. Delayed operative delivery was indicated as one of the avoidable factors. The almost non-use of instrumental vaginal delivery did also contribute to delays during the second stage of labour resulting in birth asphyxia and perinatal death. During the study period there was major renovation of the obstetric theatre and only one operating room was available. This might also have contributed to the delays in care.

In the prevention of perinatal deaths significant areas remain for health care improvements and this audit highlights the importance of care during labour and delivery. According to the findings, about one in 4 perinatal deaths in this tertiary centre could be attributed to avoidable factors linked to obstetric care. The most important policies to implement in the future are adequate foetal and maternal monitoring during labour, shortening the interval between decision and caesarean section, performing instrumental vaginal delivery when indicated, reducing prolonged labour at MNH as well as in referring hospitals and promote prompt hospital transfer after decision to refer and helping the baby to breath by immediate resuscitation at delivery. Another example of suboptimal practise is that eclampsia patients were induced with high dose of misoprostol without pre-induction assessment whether the foetus was compromised in relation to growth and/or placental insufficiency, and together with lack of proper foetal heart rate monitoring this may have resulted in asphyxia and foetal demise.

The population of Dar es Salaam has kept on growing whereas the public health facilities have remained at the same level as previously. This has resulted in congestion at this large referral centre and too many patients who do not need specialized care. To improve the services at MNH establishing new public maternities in the city is necessary. The hospital will then be able to care better for patients who really need specialized care.

## Intervention

For intervention purposes the following steps have been taken to improve the perinatal outcome. These steps will be followed by data collection to assess the impact.

• Continued medical education by re-training the midwife and doctors on the use of parthogram and interpretation of abnormal labour and helping asphyxiated babies to breath (Two trainings have been conducted with 60 participants each). This will improve monitoring during labour and resuscitation of asphyxiated newborn.

• Management protocols for eclampsia and other obstetrics emergences have been prepared and displayed in the wards notice boards.

• New sets (5 sets) of vacuum (ventouse) and Doppler machines have been purchased for assisted deliveries and assessment of foetal heart beats.

• Nurses/midwives have started routine continued medical education every morning once a week.

• A decision operation interval is checked by record tracing of the patient from the labour ward to theatre. (A log book has been opened for this purpose to identify areas of delay).

• The administration has been in contact with the municipal hospitals to streamline referrals so that there are no delays.

• An audit committee has been set up. Regular perinatal audits have been introduced every last Tuesday of the month there is a departmental perinatal audit meeting. Daily assessments of all perinatal deaths by the team on call. Weekly assessments by the audit team

• Documentation has been stressed and a new slogan not documented not done has been put in place.

• An obstetrician on call has been station in the labour ward to assist on evaluation and decision making.

## Conclusion

Regular perinatal audits to identify avoidable causes of perinatal deaths as well as feed back to the health care providers has the potential to reduce perinatal mortality in this large African urban hospital by 25%. However, this should involve the hospital management and the municipal hospitals in the city that are the referring hospitals. Priority interventions are proper monitoring of patients during labour, assessment of the foetus before decision of route of delivery, early referrals and prompt instrumental and surgical intervention. Training on monitoring labour and newborn resuscitation to newly employed midwives and doctors shall be regularly conducted. Development of management guidelines will be done before reassessing the level of improvement.

## Competing interests

The authors declare that they have no competing interests.

## Authors' contributions

HLK, participated in design of the study carried out the data collection and the analyses and drafted the first and last manuscript. IM participated in design of the study, was one of the auditors and reviewed the manuscript. JVR was one of the auditors and reviewed the manuscript. ANT was one of the auditors and reviewed the manuscript. SNM participated in designing the study and supervised the data collection LN participated in the analysis of data, and development of the manuscript. GL supervised and participated in all steps of the study.

## Pre-publication history

The pre-publication history for this paper can be accessed here:


